# The Future of Children’s Health in the Genomic Era

**DOI:** 10.5041/RMMJ.10053

**Published:** 2011-07-31

**Authors:** Alan L. Schwartz

**Affiliations:** The Harriet B. Spoehrer Professor and Chairman, Department of Pediatrics, Washington University School of Medicine, St Louis, MO, USA

**Keywords:** Genomics, bioinformatics, microbiome, genetics, child/children, pediatrics

## Abstract

The effects of genomic medicine on child health promise to be profound. Medical applications will eventually include characterizing patients’ genomes to detect predictive mutations for pre-symptomatic counseling where treatment exists; to search for causes of diseases of unknown etiology, and to detect carriers for prenatal counseling; to define cancer and other disease-based genomes to design individualized therapy; and to understand our microbiomes to modify these in health and disease. Rapid advances in technology and bioinformatics have reduced the cost and the time and increased the accuracy necessary to sequence whole genomes or whole exomes. However, complete understanding of disease will also require correlation of genomic information with high-quality phenotypic data. In addition, several critical ethical, psycho-social, and public policy issues will require clarity in the coming years. Ultimately these advances will improve the effectiveness of health care for children and for society.

For the past century, each generation of pediatricians has recognized and embraced the opportunities available to enhance the health of children. Each generation has said, “Never in the past have the opportunities to impact the lives of today’s and tomorrow’s children been so great”: they have included hygiene, public health, nutrition, vitamins, vaccines, drugs, such as antibiotics and insulin, pediatric surgery, neonatal care, etc. Today we are on the brink of another monumental change in pediatric medicine. It is far-reaching, and its implications are only now being revealed the effects of genomic medicine on child health.

Now a decade after the publication of the first draft of a reference human genome sequence,^1^–^3^ genomics has become a mainstay of biomedical research and promises to become a central pillar in understanding health and disease, especially child health and disease. Twenty-five years ago, biologists debated the value of sequencing the human genome. Today, young scientists struggle to imagine the nature of research in the antediluvian era, before the flood of genomic data.^4^ Already contributions of genomics for improving human health have come from understanding the molecular basis of inherited disease, cancer, to name just a few.

What do we mean by genomics? Genomics evolved from genetics, molecular biology, and bioinformatics. The *Annual Review of Genetics* began in 1967. The *Annual Review of Genomics and Human Genetics* began in 2000. Genomics aims to generate complete data sets, for example the entire genetic sequence complete with modifications of the cellular proteome. The generation of comprehensive data sets requires large-scale efforts which include complex organization often involving large interdisciplinary consortia, robust data standards to insure high-quality data, and sophisticated computational power. Genomics requires high-throughput low-cost data production and rapid-data release via large data catalogs and analytic tools as community resources.

The greatest impact of genomics has been the ability to investigate biological phenomena in a comprehensive, unbiased, hypothesis-free manner. In basic biology, it has reshaped our view of genome physiology, including the roles of protein-coding genes, non-coding RNAs, and regulatory sequences.

One ultimate goal for genomics is for sequencing to become so simple and inexpensive that it can be routinely deployed as a general purpose tool throughout biomedicine. Medical applications will eventually include 1) characterizing patients’ genomes to detect predictive mutations for pre-symptomatic counseling where treatment exists, to search for causes of diseases of unknown etiology, and to detect carriers for prenatal counseling; 2) cancer genomes to design individualized, optimized therapy; and 3) microbiomes to modify these in health and disease.^4^

The human genome is known to contain only ∼21,000 distinct protein-coding genes. Given the propensity of alternative splicing variants which occur in > 90% of these, there likely exist > 100,000 proteins each of which undergoes innumerable post-translational modifications, resulting in hundreds of thousands of unique proteins.

More than 98% of the genome is not contained within the protein-coding genes yet contains critical regulatory systems – for example, non-protein-coding RNAs. Among these are the recently (in 2000) discovered microRNAs which bind target mRNAs and decrease their stability. Each of the ∼100 microRNAs in the genome affect ∼200 target mRNAs, many of which are involved in key processes of development. The regions of the genome which are functionally active contain a host of “epigenomic” modifications which are layered in the core genome sequence and guide translation and physiology. It is estimated that hundreds of thousands of epigenomic modifications occur across the genome. Ultimately, a comprehensive catalog across cell types and physiologies will need to define the protein-coding and non-coding transcripts, epigenomic modifications, and all the interactions among DNA, RNA, and protein and the rules that govern these relationships.^4^

Technology development is a key driver of genomics. Both revolutionary and evolutionary technology development has fueled the remark-able increase in throughput (quantity and quality) and reduction in cost of DNA sequencing as described below. However, the inherent complexity of biology means that current technology is still not adequate for interpreting the next generation of genomic data. While many questions at the level of the individual patient may be adequately addressed today, or at least soon, we will be progressively challenged on the data analysis side perhaps more than on the data generation side.

Eric Green, Director of the National Human Genomic Research Institute at NIH in the United States, has recently written an illuminating “Perspective” in *Nature*, entitled “Charting a course for genomic medicine from base pairs to bedside”.^5^ He has approached this overview by focusing on accomplishments across five domains of genomic research: 1) understanding the structure of genomes, 2) understanding the biology of genomics, 3) understanding the biology of disease, 4) advancing the science of medicine, and 5) improving the effectiveness of health care (Figure 1).

**Figure 1:**
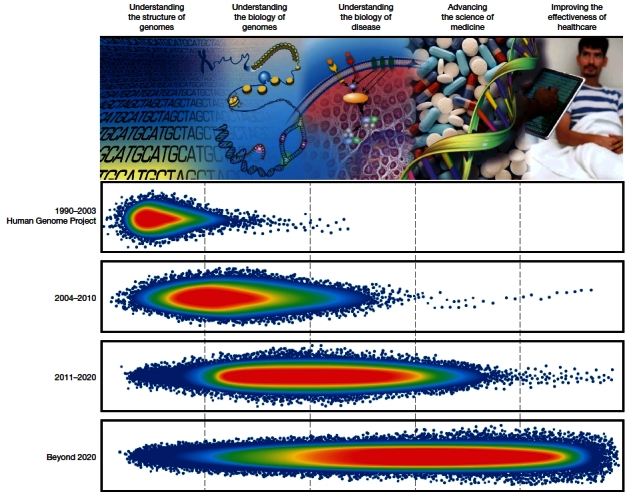
Schematic representation of accomplishments across five domains of genomics research. The progression from understanding the structure of genomes to improving the effectiveness of health care in five sequential, overlapping domains is indicated along the top. Genomic accomplishments across the domains are portrayed by hypothetical, highly schematized density plots (each blue dot reflecting a single research accomplishment, with green, yellow, and red areas reflecting sequentially higher density of accomplishments). Separate plots are shown for four time intervals: The human genome project (1990–2003), the period 2004–2010 described in the 2003 vision of the National Human Genome Research Institute (NHGRI), the period 2011–2020 described in the article by Green et al,^5^ and the open-ended future beyond 2020. Figure included with permission from Nature Publishing Group (Green ED, Guyer MS, National Human Genome Research Institute. Nature 2011;470:206).^5^

Let us examine a few areas. In terms of genome structure, today, state-of-the-art sequencing (so-called “next-generation sequencing”) can generate ∼250 billion nucleotides per single 24-h run of an Illumina HiSeq 2000 machine at a cost of ∼$25,000. The error rate is approximately 1 in 10,000. The cost for an entire human genome today is nearing $10,000 (Figure 2).

**Figure 2:**
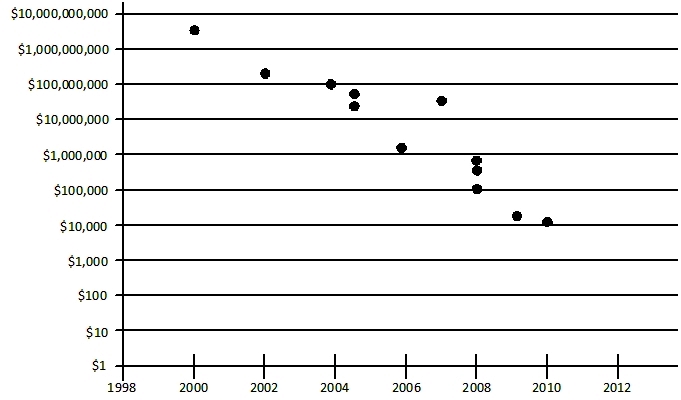
The cost of sequencing a human genome. From 2001 to the present, the cost of sequencing a complete human genome has fallen dramatically. (Adapted from R. Gibbs, Baylor College of Medicine).

Orders-of-magnitude improvements in throughput, cost-efficiency, accuracy, sensitivity, and selectivity of genomic technologies will continue to require novel approaches. Massively parallel DNA sequencing has enabled many orders of magnitude reduction in the cost of sequencing (Figure 3).^6^

**Figure 3:**
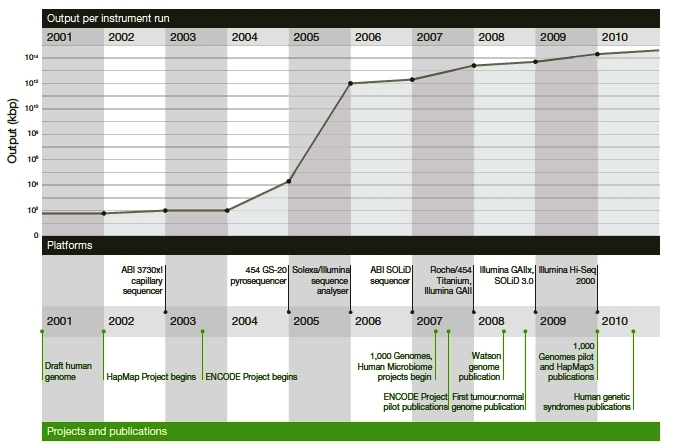
Changes in instrument capacity over the past decade. From 2001 to the present, the nucleotide sequencing output (kpp) per instrument run (*y*-axis, shown in logs) has increased dramatically. Figure included with permission (Mardis ER. Nature 2011;470:199).^6^

Today whole human genomes from single individuals are being sequenced. One recent example is the complete sequence of the first cancer genome. This was carried out by Tim Ley, Rick Wilson, and colleagues at the Washington University Genome Center in 2008.^7^ Using massively parallel sequencing technology, they sequenced the DNA of acute myelogenous leukemia (AML) and of skin cells from the same patient; the tumor genome with 33-fold coverage (98 billion bases) and for the normal skin sample 14-fold coverage (42 billion bases). Of the 2,647,695 single nucleotide variants in the tumor genome, 2,584,418 (97.6%) were in the patient’s skin genome. They ultimately identified in the tumor sample 10 genes with acquired mutations; 2 were previously known and associated with tumor progression, whereas 8 were new mutations. These 8 were present in all tumor cells at presentation (and at relapse). Their function was unknown. This study thus demonstrated the feasibility of performing whole-genome sequencing as an unbiased method for discovering cancer-imitating mutations in previously unidentified genes. The cost of this was ∼$1 million.

More recently, this same group identified recurring mutations by sequencing 188 additional AML genomes.^8^ Among those, one of the mutations was in the isocitrate dehydrogenase 1 (IDH1) gene and was present in 15 of the 188 patients. IDH1 had been previously thought to be a tumor suppressor gene. Indeed, current studies are evaluating IDH1 and other recently identified mutations as prognostic markers.

Results of this type from cancer genome sequencing will allow the development and evaluation of predictive models of cancer development, as seen here for AML (Figure 4).^9^

**Figure 4:**
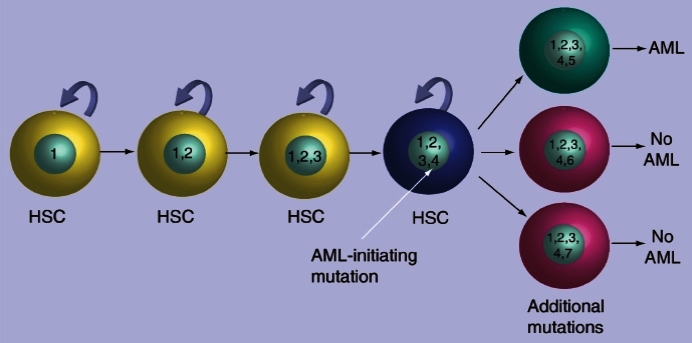
Model for evolution of genetic changes in acute myeloid leukemia. Long-lived hematopoietic stem cells (HSC) acquire a number of benign mutations (1,2,3) that do not alter the function of these cells during the life of the individual. Even though most of them are irrelevant, they are all present in the individual cell when it acquires the critical mutation (4) that sets the cancer in motion. Additional mutations (5) then cause the transformed cell to progress to overt leukemia; AML in this case. Figure included with permission (Walter MJ, et al. Per Med 2009;6:653).^9^

At present, the Washington University Genome Center in collaboration with St Jude Children’s Research Hospital is sequencing the genomes of 60 pediatric cancer genomes within 3 years.

We are now entering a period of exponential growth in cancer gene discovery that will provide many novel therapeutic targets to a large number of cancer types. Establishing the pathophysiologic relevance of individual mutations is a major challenge that must be solved. However, after thousands of cancer genomes have been sequenced, the genetic rules of cancer will become known, and new approaches for diagnosis, risk stratification, and individualized treatment will surely follow.

The international community has organized a massive cancer genome effort: The International Cancer Genome Consortium (ICGC).^10^ The ICGC was launched to co-ordinate large-scale cancer genome studies of over 25,000 cancer genomes at the genomic, epigenomic, and transcriptional levels so as to provide a platform for prognosis, therapeutic management, and development of new therapies.

While whole human genomes are being sequenced today, this remains too expensive for most human disease studies.

Traditionally Mendelian disorders have been identified via positional cloning, physical mapping, and/or candidate gene sequencing. The very recent advance of whole exome sequencing combined with bioinformatic filtering of the data set has now become a reality in which analysis of only a small number of individuals can yield the genomic defect. Whole exome sequencing is sequencing only the 2% of the genome containing the protein-encoding genes. An excellent example of this is the discovery by Bamshad (a pediatric geneticist) and colleagues of the cause of Miller’s syndrome.^11^ Miller’s syndrome, postaxial acrofacial dysostosis, is a rare syndrome with absent digits, ocular abnormalities, and cleft palates.

Ng et al.^11^ sequenced the exomes of four individuals with Miller’s syndrome, including two siblings. They used targeted exome capture of 164,000 targets, massively parallel sequencing at ∼40-fold coverage, and a step-wise bioinformatics filter scheme. This resulted in identification of only one gene, DHODH, an enzyme in pyrimidine *de-novo* biosynthesis. The four individuals with Miller’s syndrome had six rare variants in DHODH.^12^

Ng et al.^11^ conclude that whole exome sequencing of a small number of unrelated affected individuals is a powerful, efficient strategy for identifying the gene underlying rare Mendelian disorders and will likely transform the genetic analysis of monogenic traits.

However, even in the case of well understood coding regions such as exons, sequencing errors complicate downstream analyses. Current sequencing error rates hinder reliable analysis of the remaining poorly understood 98% of the genome. Obviously, very low cost, extremely accurate sequencing is essential as this becomes more common for routine clinical use.

Identifying rare variants may also utilize genotyping of large populations of individuals either sequentially (e.g. the 1,000 Genomes Project) or, to minimize cost and time, as a pooled sample. However, until recently it has been difficult to quantify the prevalence of deleterious alleles in pooled samples. Sanger and array-based resequencing are expensive for the amount of sequencing coverage obtained, as described above, and are thus incompatible with large DNA pools. Second-generation sequencing has lowered sequencing costs by over 100-fold (see above), but high error rates have hindered the analysis of large pooled samples because it is difficult to distinguish rare variants from sequencing errors. Recent advances include those of Druley (a pediatric oncologist) and Mitra who, using pooled sample sequencing, resequenced 13,237 bases of each of 1,111 individuals at approximately 2% of the costs of the original analysis by Sanger sequencing. Notably this cost saving did not come at the price of sensitivity or accuracy.^13^ Thus, very soon, rapid, large-scale sequencing will find myriad clinical uses – for example, tumor specimens upon which to plan personalized pharmacological therapy.

Similarly, recent advances have greatly accelerated our ability to understand the biology of genomes and disease, for example the information contained in the non-coding regions of DNA and the role of untranslated RNAs as described above. Among the key issues herein are: 1) defining the genetic components of disease; 2) characterization of cancer genomes; 3) developing genomics-based diagnostics; and 4) defining the role of the microbiome in health and disease.

Let us examine this last item: the role of the microbiome in health and disease. Advances in next-generation DNA sequencing have now allowed culture-independent metagenomic methods to be applied to characterization of microbial communities (i.e. microbiota) associated with human habitats, at various stages of the human life cycle and in various populations. These surveys not only define microbial organismal and genetic diversity associated with humans but begin to investigate the functional contributions that our microbes make to our physiology in health and disease. A recent study of the fecal microbiota of mono- and dizygotic twins and their mothers revealed no single identifiable abundant bacterial species was shared by all > 150 individuals examined. However, family members had a more similar microbial community structure than unrelated individuals. These and other results suggest that early environmental exposures are important determinants of microbial community structure at least within the gut.^14^

As diet and nutritional status are amongst the most important modifiable determinants of health, especially in children, and since the nutritional value of food is influenced in part by a person’s gut microbial community and its genes (i.e. the microbiome), unraveling the interrelationships between diet, gut microbiota, nutrient, and energy harvest is of great importance yet is confounded by many variables. In a recent study, Gordon and colleagues^15^ created a well defined mouse model of the human gut ecosystem by transplanting human fecal microbial communities into germ-free mice and analyzing the resultant microbial patterns temporally, spatially, and intergenerationally as well as following alteration of diet. For example, switching to a “Western” diet shifted the structure of the microbiota within 24 hours and changed the metabolic pathways and microbiome gene expression (Figure 5). These studies thus provide a platform to examine dietary targets having effects on the microbiota and/or microbiome; identify organisms which “bloom” under these conditions and study them; identify microbial-based biomarkers of health and disease; and perform prehuman therapeutic trials, etc.

**Figure 5:**
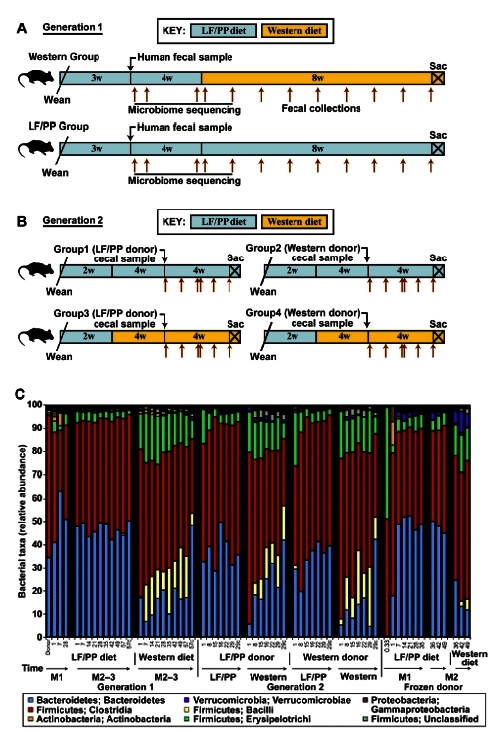
Humanized mouse gut microbiota transplant experiments. A: Design of experiments in which initial human fecal samples colonize mouse gut, adapt to various diets (low fat/plant polysaccharide (LF/PP) diet or Western diet), and are evaluated. Brown arrows indicate fecal collection time points (Generation 1). B: Reciprocal microbiota transplants in which microbiota from first-generation (A) humanized mice fed Western or LF/PP diet were transferred to LF/PP or Western-fed germ-free recipients. C: Taxonomic distribution of the bacteria in the two generation experiments described in A/B. Values represent the average relative abundance across all samples within the indicated groups following analysis of 16S rRNA gene surveys. Reproduced with permission (Turnbaugh PJ, et al. Sci Transl Med 2009;1:6ra14).^15^

In terms of genetic and non-genetic components of disease, genomics will provide a library containing the full complement of variants (both common and rare) which confer risk for inherited disease. Essential also is a complete characterization of the clinical phenotype(s) involved. Here both patients and clinicians have a critical role to play.

As Lander^4^ describes, when the human genome project was launched, ∼100 disease genes had been identified. Today ∼3,000 Mendelian disease genes are known. Even with current state-of-the-art technology and whole exome sequencing or whole genome resequencing the task is complex as a typical person has > 150 rare coding variants (as well as 2-fold more rare non-coding variants).

Most diseases are non-Mendelian and present a far greater challenge as they are polygenic. In the past 4 years > 1,000 loci affecting > 160 diseases and traits have been defined. Genome-wide association studies (GWAS) initially were focused on discovery of common variants in genes associated with common diseases. This common disease–common variant hypothesis stated that common variants (at > 1% frequency) would have a role in the etiology of common diseases. From these studies have emerged several key points:

- Most common disease/traits are influenced by a large number of variants.
- The majority of common variants have only a modest effect.
- Many more genes than previously suspected are involved.

Genome-wide association studies to date have also generally implicated hundreds of non-coding genomic regions in the pathogenesis of complex disease. These challenges have recently been summarized by Joel Hirschhorn (a pediatric endocrinologist) in a recent review in the *New England Journal of Medicine*^16^ and in the *Annual Review of Medicine*.^17^ He notes that until recently few genetic variants were known to influence reproducibly the common polygenic diseases/traits. “This relative ignorance limited potential insights into the pathophysiology of common diseases.” Skeptics have questioned the value of recent discoveries citing modest effect sizes for common variants and arguing that this would provide limited predictive value and biological insight. Hirschhorn, however, argues that the goal for these studies (e.g. GWAS) is not for the prediction of individual risk but for insight into the biological pathways involved in polygenic diseases/traits. He supports this with several examples from GWAS including: 1) studies that demonstrate that, of 23 loci associated with lipid levels, 11 implicate genes encoding lipoproteins or other key lipid metabolic steps; and 2) the identification of genes which function at the sites of action of drugs approved by the Food and Drug Administration to treat these disorders (e.g. statins and lipid levels). Importantly GWAS have highlighted pathways whose relevance to a particular disease was not suspected (for example, age-related macular degeneration and the complement system^18^).

**Table 1:** GWAS for common diseases and traits.

**Phenotype**	**Number of GWAS loci**	Proportion of heritability explained (%) *
Type 1 diabetes	41	∼60
Fetal hemoglobin levels	3	∼50
Macular degeneration	3	∼50
Type 2 diabetes	39	20–25
Crohn’s disease	71	20–25
LDL and HDL levels	95	20–25
Height	180	∼12

* Fraction of heritability explained is calculated by dividing the phenotypic variance explained by variants at loci identified by GWAS by the total heritability as inferred from epidemiological parameters. Table included with permission (Lander ES. Nature 2011;470:193).^4^ HDL, high-density lipoprotein; LDL, low-density lipoprotein.

Let us examine an example in greater detail: the current status of variants associated with the highly heritable and classic polygenic trait “height”. Height, like most common human traits and diseases, has a polygenic pattern of inheritance. DNA sequence variants at many genetic loci influence the phenotype. As noted above, genome-wide association studies have identified more than 600 variants associated with human traits, but these typically explain only small fractions of phenotypic variation, raising questions about their use in further studies. Lango et al.^19^ using 183,727 individuals have shown that hundreds of genetic variants, in at least 180 loci, influence adult height. The large number of loci reveals patterns with important implications for genetic studies of common human diseases and traits.

These loci are not random but associated with key biological pathways such as skeletal growth. The variants are often near by the causal gene. Many loci have multiple independently associated variants. Taken together these data explain ∼10% of the phenotypic variations in height. Estimates of unidentified common variants suggest that ∼20% of the heritable variation in height could be explained. Thus, detailed GWAS studies such as this can identify loci which implicate biologically relevant genes and pathways.^20^

A complete understanding of disease will require correlation of genomic information with high-quality phenotypic data. Obtaining phenotypic data which are thorough and accurate enough will require “meticulous application of phenotyping methods, improved definitions of phenotypes, new technologies, and the consistent use of data standards”.^5^

Widely accessible databases will be ne-cessary to provide the repositories for the phenotype, and genomic and environmental data sets and their linkage will be facilitated by electronic health records. The integration of genomic, phenotypic, and environmental (including pharmacological) exposure will accelerate our understanding of environmental triggers and/or modifications of disease/health. One example is the recently launched NIH-supported National Children’s Study across the U.S.

The goal of the National Children’s Study is to improve the health and well-being of children and contribute to understanding the role various factors have on health and disease. The study will examine the effects of the environment, as broadly defined to include factors such as air, water, diet, sound, family dynamics, community and cultural influences, and genetics on the growth, development, and health of children across the United States, following them from before birth until age 21 years. The study is about to commence at over 40 sites across the U.S., enroll pregnant or soon to be pregnant mothers, and follow their offspring for 21 years. The cost estimate is over $3 billion.

The National Institutes of Child Health and Human Development (NICHD), a branch of the NIH, is the major funder of child health research in the U.S., perhaps in the world. The current year’s research budget for NICHD is ∼$1.3 billion. Presently a new vision (i.e. strategic priorities) is being drafted for NICHD. Among the key themes for the future are: development, plasticity, cognitions, behavior, reproduction pregnancy, pregnancy outcomes, developmental origins of health and disease, environment, and diagnostics and therapeutics. Among the cross-cutting issues for consideration under each theme are epigenetics, personalized medicine, bioinformatics, metagenomics, and systems biology.

One broad future challenge is the complex role of human participants including children in this enterprise. The future state requires solutions to several critical interfaces with human populations/society.^5^ Areas which have and will continue to receive careful scrutiny include:

- Psycho-social issues in genomic research (e.g. issues of race/ethnicity)
- Ethical issues in genomic research (e.g. protection of human subjects)
- Psycho-social issues in genomic medicine (e.g. uncertainty of genetic predictors)
- Ethical issues in genomic medicine (e.g. direct-to-consumer marketing)
- Legal and public policy issues (e.g. regulation of genetic testing)
- Broader societal issues (e.g. gaining insights into human origins)

Ultimately, the future of genomic medicine will be to improve the lives of our patients and improve the effectiveness of health care. As noted above, the vast amounts of data, their integration, interpretation, and application will rely increasingly on electronic health records and portability.

In the U.S. today, major efforts driven by the federal government are accelerating implementation of electronic health records across health care provider platforms. While the U.S. health care system is in fact a compendium of unlinked, often independent providers (physician, hospital, etc.) there is significant movement toward high-fidelity electronic solutions to integration of the vast array of health care data for individual patients and for groups. Genomic data are one additional, although highly complex, health care data set. Among the issues to be faced is the confidentiality of the information, as well as ethical, regulatory, and legal issues.

Furthermore, a major unmet need is the ability to educate health care providers, including physicians, as well as the patients and public at large as to the utility of interpretation of and limitations of genomic information. This challenge will involve the development of paradigms which are sensitive to personal, community, and societal norms.

Next-generation sequencing and whole genome analysis are “disruptive technologies”, capable of catalyzing fundamental changes in care in pediatrics and across medicine. It is certainly within reason to anticipate that healthy individuals including new-borns and fetuses will have their genomes sequenced as the foundation of personalized programs of lifelong health promotion, disease prevention, and disease management.

Who will be the curators of genomic information over the course of an individual’s lifetime? At present, clinical genetic testing is fragmented among various specialties (clinical genetics, pediatrics, pathology, etc.). Each provides laboratory testing of one or only a few risk alleles for the “disease” of interest. In some cases, especially in the U.S., such molecular testing is offered by private companies that hold patent rights to certain genetic tests.^21^ Recently, the patenting of gene sequences has come under intense scrutiny by the U.S. Patent Office. This promises to accelerate the change in the testing landscape. Thus, it appears inevitable that the application of genome sequencing at entirely reasonable costs will undercut and likely eliminate single gene testing.^22^

The impact of genomics in medicine during the next decade and beyond will include advances in knowledge about the biology of disease, the science of medicine, and the effectiveness of health care. Indeed, current efforts directed at understanding the biology of the genome and the genetic basis for human disease will have their greatest impact decade(s) from now. Thus, one critical issue among many is the rigor with which regulatory oversight of clinical genomic testing is performed.^23^

Before I conclude, let us examine one additional robust area for child health advances in the genomic era – biofortification of nutrient crops. Elucidation of genomes of key crops (e.g. rice, wheat, corn, etc.) and their metabolic pathways has accelerated our ability to biofortify key nutrient sources. Five staple foods provide the major source of nutrition to a majority of the world’s children. Among these five is the root, cassava. Over 250 million sub-Saharan Africans rely on cassava as their major source of calories. A cassava-based diet does not provide complete nutrition. In 2005 a group of nutrition, plant biology, genetics, and public health experts initiated the BioCassava Plus project, in part supported by the Gates Foundation’s Grand Challenges. Among them is Mark Manary, pediatrician and leader in renutrition of severely malnourished children and prevention of severe malnutrition. Bio Cassava Plus is a multidisciplinary effort centered at the Danforth Plant Science Center in St Louis to “create the perfect staple plant food” by biofortifying cassava whose purpose is to prevent malnutrition. Cassava thrives in adverse climatic conditions but is devoid of protein, vitamin A, iron, and zinc. Using modern genetic engineering techniques to improve this staple tuber, cassava has recently been biofortified with ample amounts of iron, protein, and vitamin A such that the entire daily dietary requirement for these nutrients is met in a daily serving. BioCassava Plus is an extremely ambitious endeavor, the first to use successfully more than five new genes simultaneously in a crop. In late 2010, nutrient-enhanced cassava was placed in field trials in Nigeria and Kenya, with the hope of delivering better nutrition to the people of these large African nations in the coming decade.

Genomics has changed the practice of biology and medicine in fundamental ways. It has revealed the power of comprehensive views and hypothesis-free exploration to yield biological insights and medical discoveries; the value of scientific communities setting bold goals and applying team-work to accomplish them; the essential role of mathematics and computation in biomedical research; the importance of scale, process, and efficiency; the synergy between large-scale capabilities and individual creativity; and the enormous benefits of rapid and free data sharing.^4^

What will we expect from genomics for child health? Ultimately, we will have available the genetic risk for each individual fetus and child for a myriad of diseases including individual predictions for adult disease. We will have genomics-based diagnostics. We will have genetic risk assessment for drugs and other therapeutics such as replacement organs or devices. We will have key insights into how our microbiome interacts with our own genome in health and disease. We will understand how elements in the environment modify our epigenome and affect health and disease.

The future is bright, although complex. We have in our midst a revolutionary approach to advance health care for tomorrow’s children. Let us embrace the challenge and provide a better future for our patients.

## Abbreviations:

AML: acute myelogenous leukemia;
GWAS: genome-wide association studies;
ICGC: International Cancer Genome Consortium;
IDH1: isocitrate dehydrogenase 1;
NICHD: National Institutes of Child Health and Human Development.
